# Identification and Phylogenetic Analysis of a CC-NBS-LRR Encoding Gene Assigned on Chromosome 7B of Wheat

**DOI:** 10.3390/ijms140815330

**Published:** 2013-07-24

**Authors:** Caiyan Gong, Shuanghe Cao, Renchun Fan, Bo Wei, Guiping Chen, Xianping Wang, Yiwen Li, Xiangqi Zhang

**Affiliations:** 1 State Key Laboratory of Plant Cell and Chromosome Engineering, Institute of Genetics and Developmental Biology, Chinese Academy of Sciences, No.1 West Beichen Road, Chaoyang District, Beijing 100101, China; E-Mails: cygong@genetics.ac.cn (C.G.); shcao8@gmail.com (S.C.); rcfan@genetics.ac.cn (R.F.); weibo_009@genetics.ac.cn (B.W.); chenguiping968@sohu.com (G.C.); xpwang@genetics.ac.cn (X.W.); ywli@genetics.ac.cn (Y.L.); 2 Department of Life Science, Tangshan Normal University, 156 North Jianshe Road, Tangshan 063000, China

**Keywords:** wheat, NBS-LRR gene, gene mutation, molecular evolution

## Abstract

Hexaploid wheat displays limited genetic variation. As a direct A and B genome donor of hexaploid wheat, tetraploid wheat represents an important gene pool for cultivated bread wheat. Many disease resistant genes express conserved domains of the nucleotide-binding site and leucine-rich repeats (NBS-LRR). In this study, we isolated a CC-NBS-LRR gene locating on chromosome 7B from durum wheat variety Italy 363, and designated it *TdRGA-7Ba*. Its open reading frame was 4014 bp, encoding a 1337 amino acid protein with a complete NBS domain and 18 LRR repeats, sharing 44.7% identity with the PM3B protein. *TdRGA-7Ba* expression was continuously seen at low levels and was highest in leaves. *TdRGA-7Ba* has another allele *TdRGA-7Bb* with a 4 bp deletion at position +1892 in other cultivars of tetraploid wheat. In *Ae. speltoides*, as a B genome progenitor, both *TdRGA-7Ba* and *TdRGA-7Bb* were detected. In all six species of hexaploid wheats (AABBDD), only *TdRGA-7Bb* existed. Phylogenic analysis showed that all *TdRGA-7Bb* type genes were grouped in one sub-branch. We speculate that *TdRGA-7Bb* was derived from a *TdRGA-7Ba* mutation, and it happened in *Ae. speltoides*. Both types of *TdRGA-7B* participated in tetraploid wheat formation. However, only the *TdRGA-7Bb* was retained in hexaploid wheat.

## 1. Introduction

NBS-LRR genes are one of the largest families of resistance genes (R gene) in plants. They encode proteins that have a central nucleotide-binding site (NBS) and a *C*- terminal leucine-rich repeat (LRR) [1]. In the *Arabidopsis* genome there are 200 NBS-LRR class homologues [2]. In the rice genome, there are 600 NBS-LRR class homologues [3]. Based on the secondary structure of the *N*-terminus, NBS-LRR proteins are subdivided into two classes: one class carries an *N*-terminal Toll-interleukin 1 receptor (TIR) domain (TIR-NBS-LRR), and the other has a putative coiled-coil domain (CC-NBS-LRR). Only CC-NBS-LRR is present in monocotyledonous plants [4]. The function of NBS-LRR genes is to participate in plant resistance to pathogens by directly/indirectly interacting with the pathogen’s effectors. The relatively conserved domain of NBS has ATP or GTP binding activity and plays a significant role in plant defense signaling [5]. The LRR domain is a major determinant of resistance specificity, and acts as a versatile structural framework for the formation protein-protein interactions with pathogen effectors [6].

It appears that many NBS-LRR genes are tightly linked in clusters within plant genomes. These clusters of genes and the repeat structure in the LRRs domain provide a greater possibility for recombination and gene conversion, and contribute to a faster generation of novel resistance alleles. At the same time, some pseudogenes are produced by recombination or mutation, but they still have the NBS structure and also are expressed in the plant before accumulating enough mutation in their promoter. Most of them are expressed constitutively at a very low level with a variety of tissue specificities and are not induced by treatment with defense signals [7].

Disease responses caused by the NBS-LRR gene change plant metabolism and consume high energy levels [8]. It is also believed that there are fitness costs associated with the expression of NBS-LRR genes and activation of defense response pathways in the absence of pathogens [9]. It has often been observed that activation of a defense-related gene caused a defect in plant growth [10,11]. Some of the NBS-LRR genes might lose function due to mutations in the absence of pathogenic stress situations; non-functional genes can also promote new functional genes through intragenic recombination [12]. Population genetic studies showed that due to the balancing selection mechanism, NBS-LRR genes and its mutant forms widely existed in natural populations of plants [13]. The plant balances the penalty and the necessity of a resistance gene by death and reuse of NBS-LRR genes. NBS-LRR genes undergo alternative splicing [14]. Different splicing products collaboratively play a role in the disease resistance process [15].

Common wheat is one of the three major cereal crops of the world and feeds about 40% of the world population [16]. There are diploid, tetrploid and hexaploid wheats in *Triticum*. Allohexaplioid wheat (AABBDD, 2*n* = 6x =42) includes six species: *T. spelta*, *T. macha*, *T. sphaerococcum*, *T. compactum*, *T. vavilovii*, *T. aestivum*. Tetraploid wheat (AABB) includes eight species: *T. dicoccoides*, *T. turigidum*, *T. carthlicum*, *T. dicoccum*, *T. durum*, *T. turanicum*, *T. polonicum*, *T. paleocolchicum* [17]. Common wheat arose from the spontaneous hybridization of the tetraploid wheat *T. turgidum* L. (AABB, 2*n* = 4x = 28) with the diploid goatgrass *Aegilops tauschii* Coss. (DD, 2*n* = 2x =14) [18]. Tetraploid wheat arose from the spontaneous hybridization of the *Triticum urartu* (AA, 2*n* = 2x = 14) with the diploid *Aegilops speltoides* (SS, 2*n* = 2x = 14) [19]. Newly formed allopolyploids are often characterized by limited genetic variation, which is called “polyploidy bottleneck” [20]. However, R genes are expected to be variable to cope with rapidly evolving pathogens. Tetraploid wheat can be use as a gene pool for common wheat.

There are many reports about cloning plant NBS-LRR genes, functional analysis, genomic distribution, and phylogeny analysis [21–23]. However, analysis of wheat NBS-LRR genes focuses on important functional resistance gene cloning [24–26]. It still remains largely unknown about the structure and evolution of NBS-LRR genes in wheat. In this paper, we cloned an NBS-LRR gene *TdRGA-7Ba* from tetraploid wheat Italy 363. Analysis of the sequence of *TdRGA-7B* from different ploidy wheats showed that it was greatly narrowed down in polymorphism during allopolyploidization.

## 2. Results

### 2.1. Amplification and Cloning of *TdRGA-7Ba* from Italy 363

Using a pair of primers PM3b-1880F and Pm3b-3040R, a band of approximately 750 bp was amplified by PCR assay using the cDNA of Italy 363. The fragment was inserted into PGEM-T cloning vector, and twenty clones were subsequently sequenced. A homology search was carried out for these sequences using the nucleotide BLAST search available from NCBI. One sequence was found to have >90% sequence similarity with the *Pm3* like genes. In this paper, we focused only on this sequence and named it as *TdRGA-7Ba*.

**Figure 1:**
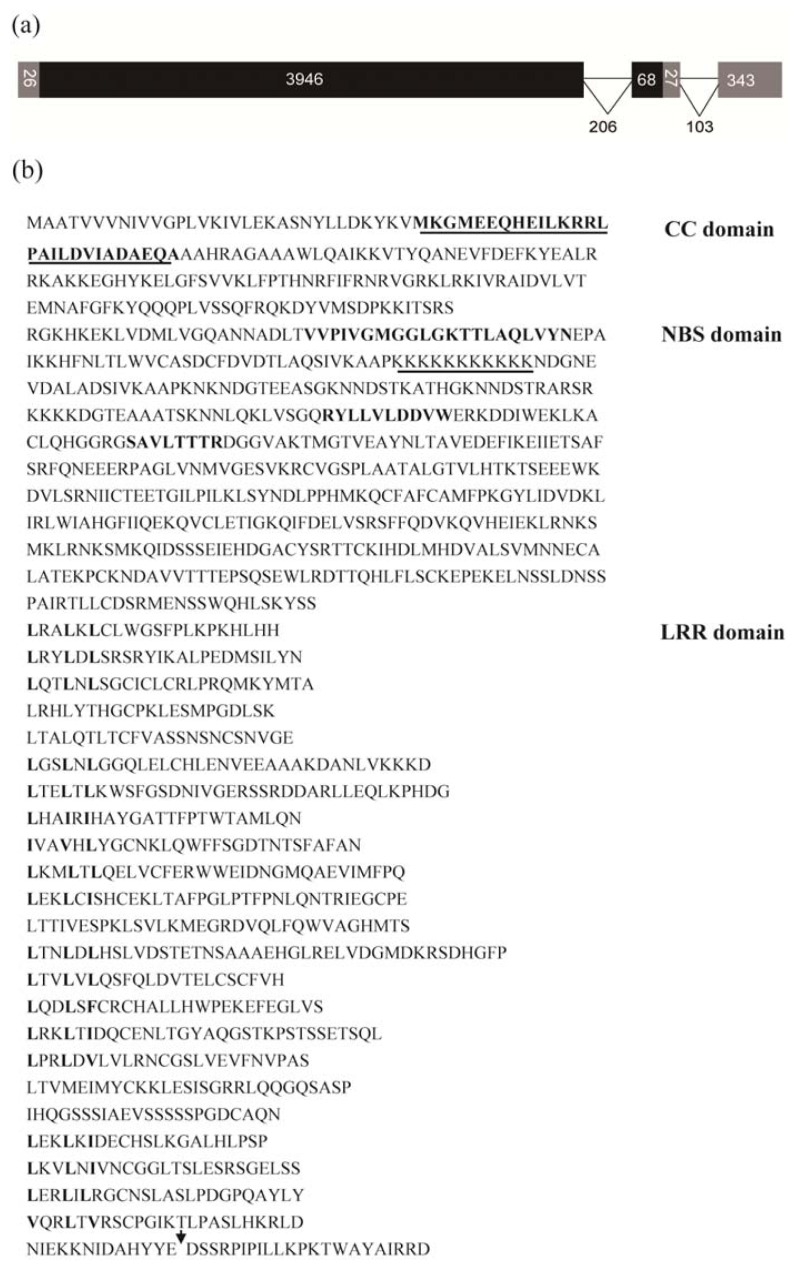
*TdRGA-7Ba* gene structure and its amino acid sequence. (**a**) Scheme of the *TdRGA-7Ba* gene. Gray boxes indicate the UTR, black boxes represent the exons, and lines represent the introns. The numbers indicate the length of very region respectively; (**b**) Amino acid sequence encoded by *TdRGA-7Ba*. The CC domain was bold and underlined. Boldface letters in the NBS regions indicate conserved amino acid motifs of the P-loop, Kinase-2 and Kinase-3, and the underlined letters represent the SSR. Bold letters in the LRR domain represent conserved amino acid residues. The arrowhead indicates the intron positions in the corresponding genomic *TdRGA-7Ba* sequence. The GenBank accession number of the *TdRGA-7Ba* sequence is KC990538.

We obtained the full-length sequence of *TdRGA-7B* using a combination of 5′-RACE and 3′-RACE (Figure 1a). The *TdRGA-7Ba* gene ORF extends 4014 bp long, and has a GC content of 46%. It encodes a protein of 1336 amino acids. As compared with the cDNA sequence, the *TdRGA-7Ba* gene consists of 3946 bp and 68 bp exons and a 206 bp intron from the start to the stop codon plus a 26 bp 5′ UTR and a 370 bp 3′ UTR. At 27 bp after the position of the stop codon, there is a 103 bp intron in the 3′ UTR. Blast analysis revealed that the amino acid sequence of *TdRGA-7Ba* had high similarity with other NBS-LRR proteins. It shared 44.7% identity over wheat powdery mildew resistance protein of PM3B (AAQ96158), and 16.0% identity to rice bacterial blight resistance protein XA1 (BAA250 68), and 15.5% homology with the *Arabidoposis Pseudomonas syringae* resistance protein RPM1 (NP187360). Analysis by the protein prediction websites InterProScan (http://www.ebi.ac.uk/Tools/InterProScan) and Pfam (http://pfam.sanger.ac.uk/search) revealed that TdRGA-7BA contained the full NBS domain Kinase 1a, Kinase 2 and Kinase 3 at the central part, and 18 LRR repeats at the *C*-terminal part. Analysis by the COILs software program (http://www.ch.embnet.org/software/COILS_form.html) revealed that there was a coiled-coil domain at the *N*-terminus. Therefore, *TdRGA-7Ba* is a CC-NBS-LRR gene (Figure 1b).

### 2.2. Expression Analysis of the *TdRGA-7Ba*

To detect the expression pattern of the *TdRGA-7Ba* in Italy 363, primer pair R-EX-F and R-EX-R were designed to amplify gene products from Italy 363 cDNA, and the PCR products were then cloned in the TA-vector and sequenced. Sequence analysis showed that the products had only one single sequence type and was not different from the original sequence. This result suggested that the primer pair could test the expression levels of *TdRGA-7Ba*.

**Figure 2:**
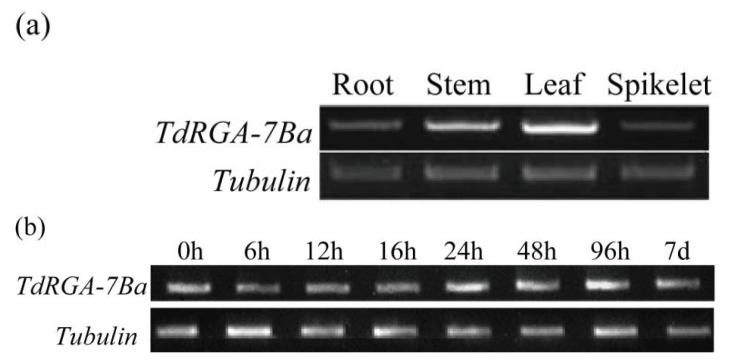
Expression patterns of *TdRGA-7Ba* in Italy 363 produced by semi-quantitative PCR. (**a**) Expression pattern in the different organs; (**b**) Expression pattern at the different time-points from 0 h to 7 day after inoculated by the *Erysiphe graminis f.* sp. *Tritici.* isolate E18. The *tubulin* gene of wheat was used as the internal control.

Transcription levels showed that *TdRGA-7Ba* was present in all tested organs: root, leaf, culm and spikelet, but was expressed at higher level in the leaf, and at lower levels in the root and spikelet (Figure 2a). One week seedlings of Italy 363 were inoculated with the powdery mildew isolate E18, and harvested for RNA isolation at 0, 6, 12, 16, 24, 48, 96 h and 7 days later. Expression tests showed there was no difference in expression between the samples (Figure 2b). This observation indicated that *TdRGA-7Ba* was not induced by the powdery mildew isolate E18. It resembled most NBS-LRR genes, which were not induced by treatment with defense signals [7].

### 2.3. Chromosomal Assignment of *TdRGA-7B* Gene

**Figure 3:**
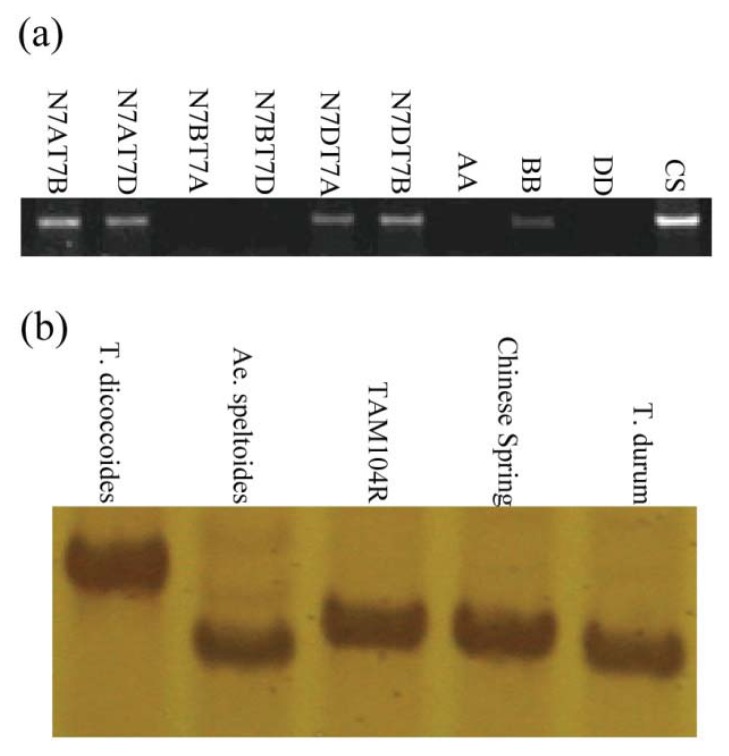
(**a**) Chromosome location of *TdRGA-7B* gene. Specific PCR products of the *TdRGA-7B* in Chinese Spring nulli-tetrasomic lines and the donors of the sub-genomes, AA: *T. urartu*; BB: *Ae. speltoides;* DD: *Ae. taurchii*; CS: Chinese Spring; (**b**) The length polymorphism of the *TdRGA-7B* SSR marker in different materials. *T. dicoccoides*: PI352322, 16 AAG repeats; *Ae. speltoides*: RM132, 8 AAG repeats; TAM104R: 13 AAG repeats; Chinese Spring: 12 AAG repeats; *T. durum*: Italy 363, 11 AAG repeats.

To determine the chromosomal location of the *TdRGA-7B* sequence we amplified the specific band from genomic DNA of the diploid wheat and *Aegilops speltoides* using the primer pair of R-EX-F and R-EX-R. Only the B genome source of *Aegilops speltoides* could amplify the band. All the nulli-tetrasomic (NT) lines of Chinese Spring could amplify the band except Nulli-7B lines. The result showed that *TdRGA-7B* was located on chromosome 7B (Figure 3a).

### 2.4. Alternative Splicing of *TdRGA-7Ba*

**Figure 4:**
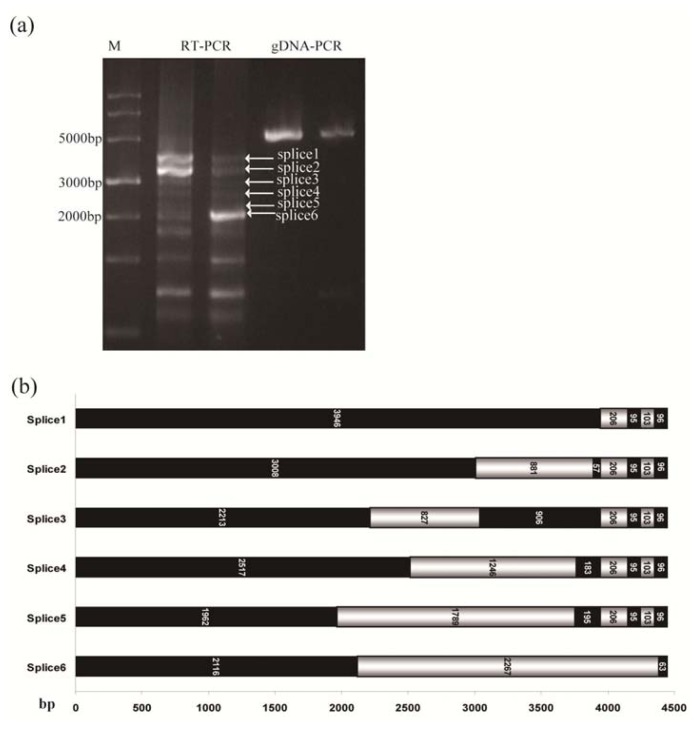
Alternative splicing of *TdRGA-7Ba*. (**a**) M: DNA marker; RT-PCR: amplified from cDNA of Italy 363 leaves, The first lane is the first round PCR, and the second lane is the second round PCR using diluted the first round PCR product as template; gDNA-PCR: amplified from DNA; (**b**) X indicates the length of splicing; Y indicates the alternative splice variants. Black boxes represent exons, and grey boxes represent introns. The numbers in the boxes represent the length of every extron or intron.

When we amplified the full length of *TdRGA-7Ba* by using the primers RLF and RLR from cDNA of Italy 363, we found several short bands on the agarose gel (Figure 4a). All about 10 bands were cloned and sequenced, and six different lengths of fragments represented *TdRGA-7Ba* gene’s different splice variants. The longest fragment was 4137-bp and the shortest was 2179-bp (from the start codon to 226-bp after the stop codon, and included the intron in the 3′UTR) (Figure 4b). All the fragments included the CC and NBS domains, but the LRR domain varied for 0 to 18 repeats.

### 2.5. Genetic Variation and Phylogenetic Analysis of *TdRGA-7B*

**Figure 5:**
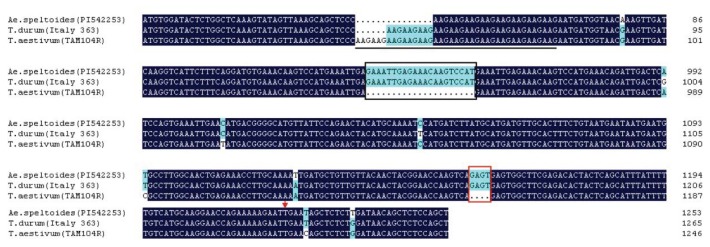
The main differences of *TdRGA-7B* in various species. The DNA sequences of *TdRAG-7B* are shown by this comparative analysis between *T. speltoides, T. durum* and *T. aestivum*. Simple sequence repeats of “AAG” are underlined, deleted nucleotides in *TdRGA-7Bb* are boxed in black (for 21 bp) and red (for 4 bp), and red arrowhead indicates the premature stop codon in *TdRGA-7Bb*.

In order to analyze the variation of the *TdRGA-7B* gene, 21 accessions of tetraploid wheat from all eight species with the AABB genome (Table 1) were performed PCR in order to detect the *TdRGA-7B* gene sequences by using the primers R-EX-F and R-EX-R. Comparison of the genomic sequences revealed two types of variation of the *TdRGA-7B* gene in tetraploid wheat. In 14 accessions, *TdRGA-7B* gene sequences were similar to it in Italy 363, which was named the *TdRGA-7Ba* type. However, in the other 6 accessions it had a 21 bp and 4 bp deletion at position +1670 and +1892. The 4 bp deletion results in the *TdRGA-7B* an in-frame premature termination at position +1957 within the transcript, thus becoming a pseudogene, which was named the *TdRGA-7Bb* type. The 6 materials with *TdRGA-7Bb* belonged to four species: *T. dicoccoides*, *T. turanicum*, *T. durum*, *T. turanicum* (Figure 5).

**Figure 6:**
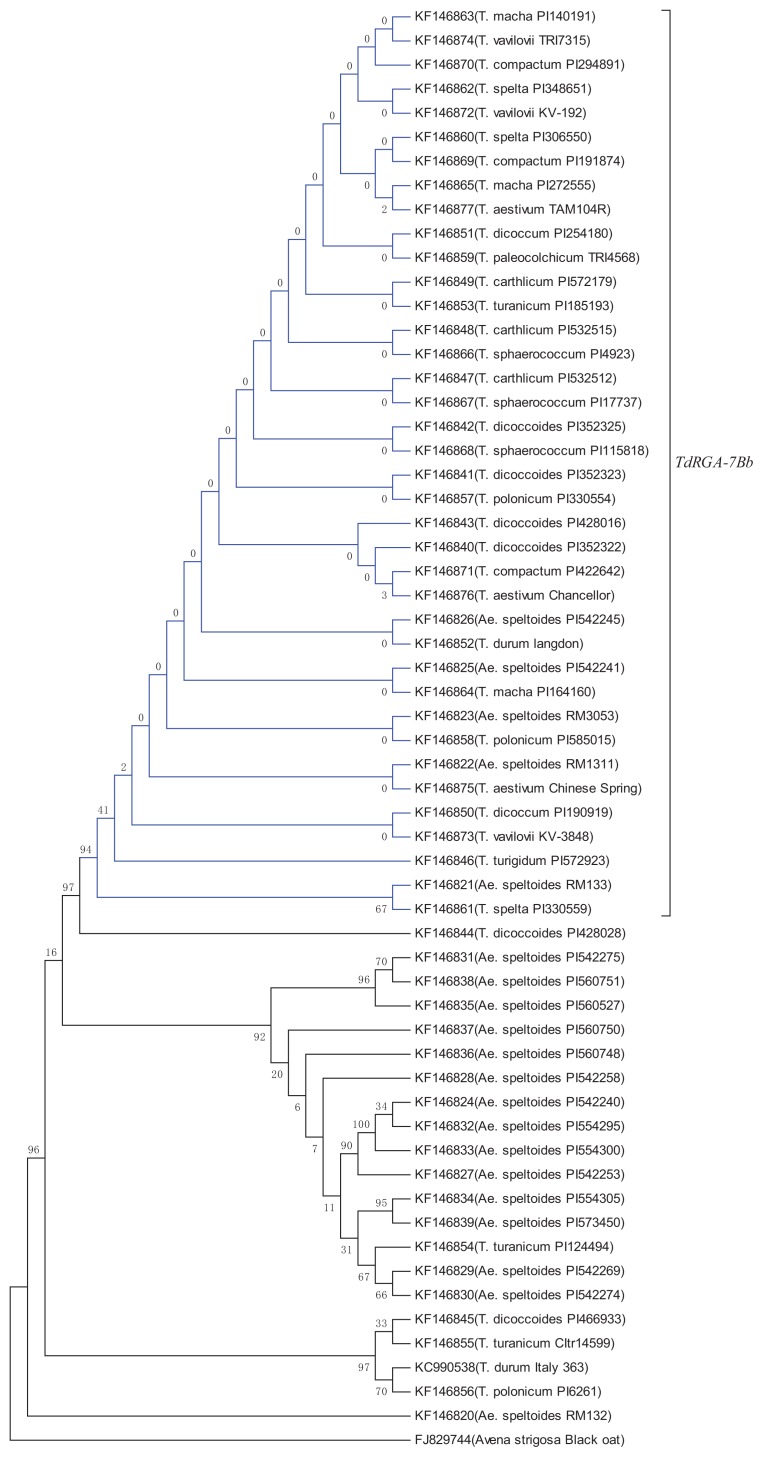
Phylogenetic relationship of *TdRGA-7B* between *Triticum* and its original species. This “maximum likelihood” phylogenetic tree derives from the 5′ region and includes the SSR and the deletion gaps of *TdRGA-7B*. All of the *TdRGA-7Bb* branches are blue. Black oat sequence is used as the out-group.

In order to further study the origin and evolution of the *TdRGA-7B* gene and the distribution in different ploidy wheat species, we detected *TdRGA-7B* variation in 20 accessions of the B genome donor *Ae. speltoides* and 18 accessions of hexaploid wheat. In *Ae. speltoides*, we found *TdRGA-7Ba* (16 accessions) and *TdRGA-7Bb* (4 accessions), but in 18 accessions representing all 6 species with the AABBDD genome of hexaploid wheat, all the samples were shown to be of the *TdRGA-7Bb* type. A phylogenic tree was constructed using MEGA5.1 software. These sequences were constructed with a black oat sequence (FJ829744) as the out-group, which was the most similar sequence with *TdRGA-7B* blasted in the NCBI program. The phylogenic tree indicated that *TdRGA-7Ba* genotypes were more divergent, while all *TdRGA-7Bb* genotypes were highly similar (Figure 6). Therefore, the *TdRGA-7Bb* type might have emerged relatively late in the process of evolution. In other words, it came from a deletion event in the *TdRGA-7Ba* gene.

### 2.6. The Development SSR Molecular Marker for *TdRGA-7B*

In the NBS domain of *TdRGA-7B* a trinucleotide repeat of AAG was different from 8 to 16 times in our materials. It can be used as gene-derived Simple Sequence Repeat (SSR) marker to track this gene. A pair of SSR primers R7B-SSR-F and R7B-SSR-R was designed based on the end sequence of the AAG repeats by on-line software primer 3.0. It could amplify a band from 422 to 446 bp length in different wheat materials (Figure 3b).

## 3. Discussion

In this paper, we have cloned an NBS-LRR gene *TdRGA-7B* from tetraploid wheat Italy 363. It was located on chromosome 7B. There were many R genes assigned on chromosome 7B; such as powdery mildew resistance genes *Pm5* [27–30] and *Pm47* [31], yellow rust resistance genes *Yr2* [32] and *Yr6* [33], stem rust resistance gene *Sr17* and leaf rust resistance gene *Lr14* [34]. Therefore, in chromosome 7B of wheat, there might be an enrichment area of resistance genes. *TdRGA-7B* may be one of the resistance genes or located near those genes. We developed an SSR marker according to the *TdRGA-7B* sequence. The SSR marker can be used as a co-dominant marker in tracking itself and those genes near *TdRGA-7B*.

In eukaryotes, alternative splicing (AS) contributes to the complexity and the diversity of gene expression [35]. Alternative splicing has been investigated more comprehensively in human and animals. About 70%–80% of genes of humans have alternative splicing shown by microarray assay [36]. It may change protein domain organization, protein activity and localization and might influence the interaction between protein subunits and protein post-transcriptional regulation. AS might also produce non-functional proteins [37]. NBS-LRR genes have been reported to undergo alternative splicing [14]. Some of the different splicing products collaboratively played a role in the disease resistance process [15]. In our study, *TdRGA-7Ba* had 6 different AS, and all of them included the whole CC and NBS domains. The function of these splicing variants need further work to prove.

Hexaploid wheat was formed only about 10,000 years ago from a natural hybridization of tetraploid wheat with diploid goatgrass *Aegilops tauschii*. Newly formed allopolyploids are often characterized by limited genetic variation, called “polyploidy bottleneck” [20]. However, R genes are expected to be variable in their ability to cope with rapidly evolving pathogens. Tetraploid wheat can be used as a gene pool for wheat. The main kinds of R genes are conserved in the NBS domains, which offers a way to isolate these types of sequence by PCR using degenerate primers designed based on the conserved domains. Using this approach, R Gene Analogs (RGAs) have been isolated extensively, such as in soybean [38], lettuce [39], barley [40], coffee [41], sunflower [42], strawberry [43], ginger [44] and cucumber [45]. In wheat, an *Mla* homologue *TaMla1* was cloned from *Triticum monococcum* and was proved to have the conserved function against powdery mildew [46]. Many cloned RGAs are either closely linked to known R gene loci or arranged in clusters similar to R genes. However, few were focused on their evolution.

We tested the *TdRGA-7B* in dipoid, tetraploid and hexaploid wheat. Both the *TdRGA-7Ba* and *TdRGA-7Bb* types were detected in *Ae. speltoides*, which showed that the differentiation between *TdRGA-7Ba* and *TdRGA-7Bb* was before the formation of tetraploid wheat, which is 0.5 million years ago [47]. The phylogenic tree indicated the *TdRGA-7Bb* to be assembled in one sub-branch, so we speculate that the formation of *TdRGA-7Bb* was the result of one single mutation. The fact that two types of *TdRGA-7B* are in the tetraploid wheat, demonstrates that both types of *TdRGA-7B* participated in the formation of tetraploid wheat. That is to say, at least two independent hybridization events happened at that time. However, only the *TdRGA-7Bb* type is in hexaploid wheat (AABBDD). This suggests that maybe only the *TdRGA-7Bb* participated in the formation of hexaploid wheat, or *TdRGA-7Ba* also participated to the process and then has been lost.

The reason of only *TdRGA-7Bb* existing in hexaploid wheat is speculated as follows: first, hybrid incompatibility. One of the hybrid incompatibilities is hybrid necrosis, and the resistance process to pathogen is in the content of hypersensitive necrosis. The resistance genes can induce necrosis in the hybrid as its by-product. Resistance genes recognize effectors of pathogen, so it is highly more likely to block the distant hybridization than other proteins by recognizing foreign proteins [48]. An NBS-LRR-type disease resistance (R) gene was necessary and sufficient for induction of hybrid necrosis in intraspecific crosses of *Arabidopsis thaliana* [49]. The functional *TdRGA-7Ba* might block the hybridization of tetraploid wheat and goatgrass and was excluded out of the hexaploid wheat. Second, fitness costs. Constitutively expressed NBS-LRR genes are not particularly useful in an environment without the existence of a counterpart pathogen, and hexaploid wheat often has gene functional redundancy because of tripled genomes. These genes tend to be lost or become pseudogenes to avoid a fitness cost to the host species [50]. *TdRGA-7Ba* might have lost its value in hexaploid wheat and was thus evolutionarily excluded.

## 4. Experimental Section

### 4.1. Plant Materials T. durum

Italy 363 was kindly provided by Dr. Fangpu Han, Institute of Genetics and Developmental Biology, Chinese Academy of Sciences (Beijing, China). The wheat line Chancellor and the powdery mildew isolate E18 were kindly supplied by Dr. Xiayu Duan, Insitute of Plant Protection, Chinese Academy of Agricultural Sciences (Beijing, China). All other diploid, tetroploid and haxploid wheats (Table 1), *Aegilops speltoides* (the source of the B genome), diploid goatgrass *Aegilops tauschii* (the source of D genome) and the nulli-tetrasomic (NT) lines of Chinese Spring (CS) were collected by State Key Laboratory of Plant Cell and Chromosome Engineering (Beijing, China).

### 4.2. DNA, RNA Extraction and cDNA Synthesis

All wheat seedlings were grown in a growth chamber under a 16 h/8 h, 20 °C/18 °C day/night cycle with 70% relative humidity. The one-week seedlings from all plant materials were harvested, frozen immediately in liquid nitrogen, and stored at −80 °C. Genomic DNA was extracted by the CTAB method [51]. Total RNA was extracted from leaves and other organs by TRIZOL (Invitrogen, Carlsbad, CA, USA) following the manufacturer’s instruction. The first strand cDNA was reverse transcribed using oligo(dT)18 primers (TaKaRa, Shiga, Japan) and transcriptase (M-MLV, Promega, Madison, WI, USA) at 42 °C for 1.5 h. Control reactions included a positive RT-PCR control with tubulin specific primers (tubF**-**345: 5′-TGAGGACTG GTGCTTACCGC-3′ and tubR-852: 5′-GCACCATCAAACCTCAGGGA-3′, which were designed according to *Triticum aestivum alpha*-*tubulin* cDNA (TAU76558) ) and used to amplify the cDNA, and a negative control with tubulin primers, which were used to amplify the RNA to test for genomic DNA contamination.

### 4.3. *TdRGA-7Ba* Sequencing and Analysis

The *Pm3b* sequence [26] was used to design primers PM3b-1880F (5′-TCACCTAAGGTACCTTG-3′), and PM3b-3040R (5′-TTGGTGCTTCGGGTA-3′). All primers were synthesized by the Invitrogen Company (Beijing, China). PCR reactions were performed in 50 μL mixtures containing 5 μL first strand cDNA as template, 5 μL 10× PCR buffer, 4 μL dNTPs (2.5 mmol/L), 1.25 μL each primer, 0.5 μL Taq DNA polymerase and 33 μL ddH_2_O. Amplification cycling conditions were the following: 95 °C for 5 min, 35 cycles of 94 °C for 30 s, 57 °C for 30 s, 72 °C for 2 min, with a final extension at 72 °C for 10 min. PCR products were separated by 1% agarose gel electrophoresis. Fragments were purified using a Gel Extraction Kit (TianGen, Beijing, China) and cloned into the pGEM-T plasmid vector (Promega, Madison, WI, USA) for subsequent sequence analysis.

The full-length cDNA sequence of *TdRGA-7Ba* was obtained by using the SMARTer™ RACE cDNA Amplification Kit (CLONTECH, Palo Alto, CA, USA). The experiments were carried out according to the product user manual. According to the sequence of the PCR fragment, gene specific primers (GSP) were designed. 5′-RACE PCR (Rapid Amplification of cDNA Ends) was performed with the general primer UPM and 5′ GSP (5′-TCACTGAGATCCTTCTTGTTTCCAAGG-3′), and 3′-RACE with general primer UPM and 3′-GSP (5′-GTTTATGAGCAATTGTGGAAAGTTGGTAG-3′).

The RACE sequences were constructed to full length *TdRGA-7Ba* cDNA by DNAMAN (http://www.lynnon.com). According to the constructed sequence, primer pair RLF (5′-TCCTCTCCACCTTGCGAG-3′) and RLR (5′-GTGTCGCCGTGCCTCTTG-3′) were designed, and used to amplify the full length cDNA and DNA sequences from Italy 363. Conserved domain prediction in *TdRGA-7Ba* was performed using the InterProScan (http://www.ebi.ac.uk/Tools/InterProScan) and pfam (http://pfam.sanger.ac.uk/search) software programs. Coiled-coil regions were predicted by COILS (http://www.ch.embnet.org/software/COILS_form.html) software program.

### 4.4. The Expression Pattern of *TdRGA-7Ba*

The expression pattern of *TdRGA-7Ba* was tested by semi-quantitative PCR. The gene specific primer pair R-EX-F (5′- ATGTGGATACTCTGGCTC -3′) and R-EX-R (5′- AGCTGGAGAGCTGTTATCC -3′) were designed according to the coding region of *TdRGA-7Ba*. PCR products of *tubulin* (TAU76558) in wheat were used as the internal control. The volume of PCR reaction was 50 μL with 5 μL first-strand cDNA as the template. Reactions were performed with EX Taq Polymerase (TaKaRa), using the following profiles: 94 °C for 5 min, 27–32 cycles of 30 s at 94 °C, 30 s at 58 °C, 1.5 min at 72 °C, and with a final extension 72 °C for 10 min. The *tubulin* PCR assay was performed by 27 cycles and the *TdRGA-7Ba* PCR assay was 32 cycles. The PCR products were separated on 1.0% (*w*/*v*) agarose gels.

### 4.5. Chromosomal Assignment of *TdRGA-7B* Gene Sequence

The *TdRGA-7B* specific PCR band was used to test the existence of *TdRGA-7B* in Chinese Spring, *T. urartu* Thum, *Aegilops speltoides* (Tausch) Gren. and a series of Chinese Spring nulli-tetrasomic (CS-NT) lines. The PCR assay was performed by using the primers of R-EX-F and R-EX-R. The Products were separated on 1.0% (*w*/*v*) agarose gels.

### 4.6. Phylogenetic Analysis of *TdRGA-7B*

The *TdRGA-7B* fragment was amplified by the primer pair R-EX-F and R-EX-R from every material described in Table 1. Every material was performed PCR assay with tree times separately and at least three clones were sequenced from every PCR product to reduce experimental error. Phylogenetic trees were constructed from CLUSTALW alignments of the genomic DNA sequences of *TdRGA-7B* using the Maximum-Likelihood method available in the Mega5.1 software program (http://www.megasoftware.net/). Confidence values for nodes were calculated using 1000 bootstraps.

### 4.7. The Development of the SSR Molecular Marker for *TdRGA-7B*

In the NBS domain of *TdRGA-7B* a trinucleotide repeat of AAG was different from 8 to 16 times in our materials. A pair of SSR primer R7B-SSR-F (5′-GAAAGACCACGTTAGCAC-3′) and R7B-SSR-R (5′-TTCCCAAACATCATC CAG-3′) were designed based on the end sequence of the AAG repeat by using an on-line software primer 3.0. The volume of the PCR reaction samples was 20 μL with 2 μL of DNA as template. Reactions were performed with EX Taq Polymerase (TaKaRa), using the following profiles: 94 °C for 5 min, 40 cycles of 30 s at 94 °C, 30 s at 58 °C, 30 s at 72 °C, and with a final extension of 72 °C for 10 min. The products were separated on 8% polyacrylamide gels.

## 5. Conclusions

In the current work, we cloned an NBS-LRR gene *TdRGA-7Ba* from tetraploid wheat cultivar Italy 363. Analysis of the sequence of *TdRGA-7B* from different ploidy wheat showed that there were two types of *TdRGA-7B* in dipoid and tetraploid wheat, but only the mutated *TdRGA-7Bb* type existed in all six species of hexaploid wheats (AABBDD). *TdRGA-7Bb* is a mutant of *TdRGA-7Ba* and was formed in *Ae. speltoides*. Two types of *TdRGA-7B* participated in the formation of tetraploid wheat, but only the *TdRGA-7Bb* form was retained in hexaploid wheat. This gene is greatly diminished in polymorphism during allopolyploidization.

## Figures and Tables

**Table 1:** The variation of *TdRGA-7B* gene in *Ae. speltoides*, tetraploid and hexaploid wheat.

Species	Source designation	Genome	Genotype	GenBank accession No.
Ae. speltoides	RM132	BB	*TdRGA-7Ba*	KF146820
Ae. speltoides	RM133	BB	*TdRGA-7Bb*	KF146821
Ae. speltoides	RM1311	BB	*TdRGA-7Bb*	KF146822
Ae. speltoides	RM3053	BB	*TdRGA-7Bb*	KF146823
Ae. speltoides var ligustica	PI542240	BB	*TdRGA-7Ba*	KF146824
Ae. speltoides var ligustica	PI542241	BB	*TdRGA-7Bb*	KF146825
Ae. speltoides	PI542245	BB	*TdRGA-7Bb*	KF146826
Ae. speltoides var. ligustica	PI542253	BB	*TdRGA-7Ba*	KF146827
Ae. speltoides	PI542258	BB	*TdRGA-7Ba*	KF146828
Ae. speltoides	PI542269	BB	*TdRGA-7Ba*	KF146829
Ae. speltoides	PI542274	BB	*TdRGA-7Ba*	KF146830
Ae. speltoides	PI542275	BB	*TdRGA-7Ba*	KF146831
Ae. speltoides	PI554295	BB	*TdRGA-7Ba*	KF146832
Ae. speltoides	PI554300	BB	*TdRGA-7Ba*	KF146833
Ae. speltoides var ligustica	PI554305	BB	*TdRGA-7Ba*	KF146834
Ae. speltoides var ligustica	PI560527	BB	*TdRGA-7Ba*	KF146835
Ae. speltoides	PI560748	BB	*TdRGA-7Ba*	KF146836
Ae. speltoides	PI560750	BB	*TdRGA-7Ba*	KF146837
Ae. speltoides	PI560751	BB	*TdRGA-7Ba*	KF146838
Ae. speltoides	PI573450	BB	*TdRGA-7Ba*	KF146839
T. dicoccoides	PI352322	AABB	*TdRGA-7Bb*	KF146840
T. dicoccoides	PI352323	AABB	*TdRGA-7Bb*	KF146841
T. dicoccoides	PI352325	AABB	*TdRGA-7Bb*	KF146842
T. dicoccoides	PI428016	AABB	*TdRGA-7Bb*	KF146843
T. dicoccoides	PI428028	AABB	*TdRGA-7Ba*	KF146844
T. dicoccoides	PI466933	AABB	*TdRGA-7Ba*	KF146845
T. turigidum	PI572923	AABB	*TdRGA-7Bb*	KF146846
T. carthlicum	PI532512	AABB	*TdRGA-7Bb*	KF146847
T. carthlicum	PI532515	AABB	*TdRGA-7Bb*	KF146848
T. carthlicum	PI572179	AABB	*TdRGA-7Bb*	KF146849
T. dicoccum	PI190919	AABB	*TdRGA-7Bb*	KF146850
T. dicoccum	PI254180	AABB	*TdRGA-7Bb*	KF146851
T. durum	Langdon	AABB	*TdRGA-7Bb*	KF146852
T. durum	Italy 363	AABB	*TdRGA-7Ba*	KC990538
T. turanicum	PI185193	AABB	*TdRGA-7Bb*	KF146853
T. turanicum	PI124494	AABB	*TdRGA-7Ba*	KF146854
T. turanicum	CItr14599	AABB	*TdRGA-7Ba*	KF146855
T. polonicum	PI6261	AABB	*TdRGA-7Ba*	KF146856
T. polonicum	PI330554	AABB	*TdRGA-7Bb*	KF146857
T. polonicum	PI585015	AABB	*TdRGA-7Bb*	KF146858
T. paleocolchicum	TRI4568	AABB	*TdRGA-7Bb*	KF146859
T. spelta	PI306550	AABBDD	*TdRGA-7Bb*	KF146860
T. spelta	PI330559	AABBDD	*TdRGA-7Bb*	KF146861
T. spelta	PI348651	AABBDD	*TdRGA-7Bb*	KF146862
T. macha	PI140191	AABBDD	*TdRGA-7Bb*	KF146863
T. macha	PI164160	AABBDD	*TdRGA-7Bb*	KF146864
T. macha	PI272555	AABBDD	*TdRGA-7Bb*	KF146865
T. sphaerococcum	PI4923	AABBDD	*TdRGA-7Bb*	KF146866
T. sphaerococcum	PI17737	AABBDD	*TdRGA-7Bb*	KF146867
T. sphaerococcum	PI115818	AABBDD	*TdRGA-7Bb*	KF146868
T. compactum	PI191874	AABBDD	*TdRGA-7Bb*	KF146869
T. compactum	PI294891	AABBDD	*TdRGA-7Bb*	KF146870
T. compactum	PI422642	AABBDD	*TdRGA-7Bb*	KF146871
T. vavilovii	KU192	AABBDD	*TdRGA-7Bb*	KF146872
T. vavilovii	KU3848	AABBDD	*TdRGA-7Bb*	KF146873
T. vavilovii	TRI7315	AABBDD	*TdRGA-7Bb*	KF146874
T. aestivum	Chinese Spring	AABBDD	*TdRGA-7Bb*	KF146875
T. aestivum	Chancellor	AABBDD	*TdRGA-7Bb*	KF146876
T. aestivum	TAM104R	AABBDD	*TdRGA-7Bb*	KF146877

The RM accessions are from Chinese Crop Germplasm Resources Information Network, China;the CItr and PI accessions are from USDA-ARS, The Germplasm Resources Information Network (GRIN), USA; the TRI accessions are from Genebank Information System of the IPK Gatersleben, Germany; the KU accessions are from the National BioResource Project-wheat, Japan.

## References

[1] Dangl J.L., Jones J.D. (2001). Plant pathogens and integrated defence responses to infection. Nature.

[2] Meyers B.C., Kozik A., Griego A., Kuang H., Michelmore R.W. (2003). Genome-wide analysis of NBS-LRR-encoding genes in *Arabidopsis*. Plant Cell.

[3] Bai J., Pennill L.A., Ning J., Lee S.W., Ramalingam J., Webb C.A., Zhao B., Sun Q., Nelson J.C., Leach J.E. (2002). Diversity in nucleotide binding site-leucine-rich repeat genes in cereals. Genome Res.

[4] Tarr D.E., Alexander H. (2009). TIR-NBS-LRR genes are rare in monocots: Evidence from diverse monocot orders. BMC Res. Notes.

[5] Tameling W.I., Elzinga S.D., Darmin P.S., Vossen J.H., Takken F.L., Haring M.A., Cornelissen B.J. (2002). The tomato R gene products I-2 and MI-1 are functional ATP binding proteins with ATPase activity. Plant Cell.

[6] Jiang H., Wang C., Ping L., Tian D., Yang S. (2007). Pattern of LRR nucleotide variation in plant resistance genes. Plant Sci.

[7] Tan X., Meyers B.C., Kozik A., West M.A., Morgante M., St Clair D.A., Bent A.F., Michelmore R.W. (2007). Global expression analysis of nucleotide binding site-leucine rich repeat-encoding and related genes in *Arabidopsis*. BMC plant Biol.

[8] Van der Hoorn R.A., de Wit P.J., Joosten M.H. (2002). Balancing selection favors guarding resistance proteins. Trends Plant Sci.

[9] Joshi R., Nayak S. (2011). Functional characterization and signal transduction ability of nucleotide-binding site-leucine-rich repeat resistance genes in plants. Genet. Mol. Res.

[10] Grant J.J., Chini A., Basu D., Loake G.J. (2003). Targeted activation tagging of the *Arabidopsis* NBS-LRR gene, *ADR1*, conveys resistance to virulent pathogens. Mol. Plant Microbe Interact.

[11] Kato H., Shida T., Komeda Y., Saito T., Kato A. (2011). Overexpression of the activated disease resistance 1-like1 (*ADR1-L1*) gene results in a dwarf phenotype and activation of defense-related gene expression in *Arabidopsis thaliana*. J. Plant Biol.

[12] Huang L., Brooks S., Li W., Fellers J., Nelson J.C., Gill B. (2009). Evolution of new disease specificity at a simple resistance locus in a crop-weed complex: Reconstitution of the *Lr21* gene in wheat. Genetics.

[13] Meyers B.C., Kaushik S., Nandety R.S. (2005). Evolving disease resistance genes. Curr. Opin. Plant Biol.

[14] Ferrier-Cana E., Macadre C., Sevignac M., David P., Langin T., Geffroy V. (2005). Distinct post-transcriptional modifications result into seven alternative transcripts of the CC-NBS-LRR gene *JA1tr* of *Phaseolus vulgaris*. Theor. Appl. Genet.

[15] Zhang X.-C., Gassmann W. (2003). *RPS4*-mediated disease resistance requires the combined presence of *RPS4* transcripts with full-length and truncated open reading frames. Plant Cell.

[16] Gupta P., Mir R., Mohan A., Kumar J. (2008). Wheat genomics: Present status and future prospects. Int. J. Plant Genomics.

[17] Matsuoka Y. (2011). Evolution of polyploid Triticum wheats under cultivation: The role of domestication, natural hybridization and allopolyploid speciation in their diversification. Plant Cell Physiol.

[18] McFadden E., Sears E. (1946). The origin of *Triticum spelta* and its free-threshing hexaploid relatives. J. Hered.

[19] Huang S., Sirikhachornkit A., Su X., Faris J., Gill B., Haselkorn R., Gornicki P. (2002). Genes encoding plastid acetyl-CoA carboxylase and 3-phosphoglycerate kinase of the *Triticum/Aegilops* complex and the evolutionary history of polyploid wheat. Proc. Natl. Acad. Sci. USA.

[20] Feldman M., Levy A.A. (2012). Genome evolution due to allopolyploidization in wheat. Genetics.

[21] Yue J.X., Meyers B.C., Chen J.Q., Tian D., Yang S. (2012). Tracing the origin and evolutionary history of plant nucleotide-binding site-leucine-rich repeat (NBS-LRR) genes. New Phytol.

[22] Kim T.-H., Kunz H.-H., Bhattacharjee S., Hauser F., Park J., Engineer C., Liu A., Ha T., Parker J.E., Gassmann W. (2012). Natural variation in small molecule-induced TIR-NB-LRR signaling induces root growth arrest via EDS1- and PAD4-complexed R protein VICTR in *Arabidopsis*. Plant Cell.

[23] Okuyama Y., Kanzaki H., Abe A., Yoshida K., Tamiru M., Saitoh H., Fujibe T., Matsumura H., Shenton M., Galam D.C. (2011). A multifaceted genomics approach allows the isolation of the rice Pia-blast resistance gene consisting of two adjacent NBS-LRR protein genes. Plant J.

[24] De Majnik J., Ogbonnaya F.C., Moullet O., Lagudah E.S. (2003). The *Cre1* and *Cre3* nematode resistance genes are located at homeologous loci in the wheat genome. Mol. Plant Microbe Interact.

[25] Feuillet C., Travella S., Stein N., Albar L., Nublat A., Keller B. (2003). Map-based isolation of the leaf rust disease resistance gene *Lr10* from the hexaploid wheat (*Triticum aestivum* L.) genome. Proc. Natl. Acad. Sci. USA.

[26] Yahiaoui N., Srichumpa P., Dudler R., Keller B. (2004). Genome analysis at different ploidy levels allows cloning of the powdery mildew resistance gene *Pm3b* from hexaploid wheat. Plant J.

[27] Law C., Wolfe M. (1966). Location of genetic factors for mildew resistance and ear emergence time on chromosome 7B of wheat. Can. J. Genet. Cytol.

[28] Hsam S., Huang X., Zeller F. (2001). Chromosomal location of genes for resistance to powdery mildew in common wheat (*Triticum aestivum* L. em Thell.) 6. Alleles at the *Pm5* locus. Theor. Appl. Genet.

[29] Huang X., Wang L., Xu M., Röder M. (2003). Microsatellite mapping of the powdery mildew resistance gene *Pm5e* in common wheat (*Triticum aestivum* L.). Theor. Appl. Genet.

[30] Mohler V., Bauer A., Bauer C., Flath K., Schweizer G., Hartl L. (2011). Genetic analysis of powdery mildew resistance in German winter wheat cultivar Cortez. Plant Breed.

[31] Xiao M., Song F., Jiao J., Wang X., Xu H., Li H. (2013). Identification of the gene *Pm47* on chromosome 7BS conferring resistance to powdery mildew in the Chinese wheat landrace Hongyanglazi. Theor. Appl. Genet.

[32] Lin F., Xu S.C., Zhang L.J., Miao Q., Zhai Q., Li L. (2005). SSR marker of wheat stripe rust resistance gene *Yr2*. J. Tritical Crops.

[33] Li Y., Niu Y.C. (2007). Identification of molecular markers for wheat stripe rust resistance gene *Yr6*. Acta Agr. Boreali-Sinica.

[34] McIntosh R., Luig N., Baker E. (1967). Genetic and cytogenetic studies of stem rust, leaf rust, and powdery mildew resistances in Hope and related wheat cultivars. Aust. J. Biol. Sci.

[35] Sammeth M., Foissac S., Guigó R. (2008). A general definition and nomenclature for alternative splicing events. PLoS Comput. Biol.

[36] Johnson J.M., Castle J., Garrett-Engele P., Kan Z., Loerch P.M., Armour C.D., Santos R., Schadt E.E., Stoughton R., Shoemaker D.D. (2003). Genome-wide survey of human alternative pre-mRNA splicing with exon junction microarrays. Science.

[37] Simpson C.G., Fuller J., Maronova M., Kalyna M., Davidson D., McNicol J., Barta A., Brown J.W. (2008). Monitoring changes in alternative precursor messenger RNA splicing in multiple gene transcripts. Plant J.

[38] Kanazin V., Marek L.F., Shoemaker R.C. (1996). Resistance gene analogs are conserved and clustered in soybean. Proc. Natl. Acad. Sci. USA.

[39] Shen K.A., Meyers B.C., Islam-Faridi M.N., Chin D.B., Stelly D.M., Michelmore R.W. (1998). Resistance gene candidates identified by PCR with degenerate oligonucleotide primers map to clusters of resistance genes in lettuce. Mol. Plant Microbe Interact.

[40] Leister D., Kurth J., Laurie D.A., Yano M., Sasaki T., Devos K., Graner A., Schulze-Lefert P. (1998). Rapid reorganization of resistance gene homologues in cereal genomes. Proc. Natl. Acad. Sci. USA.

[41] Noir S., Combes M.-C., Anthony F., Lashermes P. (2001). Origin, diversity and evolution of NBS-type disease-resistance gene homologues in coffee trees (*Coffea* L.). Mol. Genet. Genomics.

[42] Gedil M.A., Slabaugh M.B., Berry S., Johnson R., Michelmore R., Miller J., Gulya T., Knapp S.J. (2001). Candidate disease resistance genes in sunflower cloned using conserved nucleotide-binding site motifs: Genetic mapping and linkage to the downy mildew resistance gene *Pl1*. Genome.

[43] Zamora M.M., Castagnaro A., Ricci J.D. (2004). Isolation and diversity analysis of resistance gene analogues (RGAs) from cultivated and wild strawberries. Mol. Genet. Genomics.

[44] Nair R.A., Thomas G. (2007). Evaluation of resistance gene (R-gene) specific primer sets and characterization of resistance gene candidates in ginger (*Zingiber officinale* Rosc.). Curr. Sci.

[45] Wan H., Zhao Z., Qian C., Sui Y., Malik A.A., Chen J. (2010). Selection of appropriate reference genes for gene expression studies by quantitative real-time polymerase chain reaction in cucumber. Anal. Biochem.

[46] Jordan T., Seeholzer S., Schwizer S., Töller A., Somssich I.E., Keller B. (2011). The wheat *Mla* homologue *TmMla1* exhibits an evolutionarily conserved function against powdery mildew in both wheat and barley. Plant J.

[47] Huang S., Sirikhachornkit A., Faris J.D., Su X., Gill B.S., Haselkorn R., Gornicki P. (2002). Phylogenetic analysis of the acetyl-CoA carboxylase and 3-phosphoglycerate kinase loci in wheat and other grasses. Plant Mol. Biol.

[48] Yamamoto E., Takashi T., Morinaka Y., Lin S., Wu J., Matsumoto T., Kitano H., Matsuoka M., Ashikari M. (2010). Gain of deleterious function causes an autoimmune response and Bateson-Dobzhansky-Muller incompatibility in rice. Mol. Genet. Genomics.

[49] Bomblies K., Lempe J., Epple P., Warthmann N., Lanz C., Dangl J.L., Weigel D. (2007). Autoimmune response as a mechanism for a Dobzhansky-Muller-type incompatibility syndrome in plants. PLoS Biol.

[50] Tian D., Traw M., Chen J., Kreitman M., Bergelson J. (2003). Fitness costs of R-gene-mediated resistance in *Arabidopsis thaliana*. Nature.

[51] Allen G., Flores-Vergara M., Krasynanski S., Kumar S., Thompson W. (2006). A modified protocol for rapid DNA isolation from plant tissues using cetyltrimethylammonium bromide. Nat. Protoc.

